# Artocarpin Induces Apoptosis in Human Cutaneous Squamous Cell Carcinoma HSC-1 Cells and Its Cytotoxic Activity Is Dependent on Protein-Nutrient Concentration

**DOI:** 10.1155/2015/236159

**Published:** 2015-01-14

**Authors:** Stephen Chu-Sung Hu, Chi-Ling Lin, Hui-Min Cheng, Gwo-Shing Chen, Chiang-Wen Lee, Feng-Lin Yen

**Affiliations:** ^1^Department of Dermatology, College of Medicine, Kaohsiung Medical University, Kaohsiung, Taiwan; ^2^Department of Dermatology, Kaohsiung Medical University Hospital, Kaohsiung, Taiwan; ^3^Division of Basic Medical Sciences, Department of Nursing, Chang Gung Institute of Technology and Chronic Diseases and Health Promotion Research Center, Chiayi, Taiwan; ^4^Research Center for Industry of Human Ecology, Chang Gung University of Science and Technology, Kweishan, Taoyuan 33303, Taiwan; ^5^Department of Fragrance and Cosmetic Science, College of Pharmacy, Kaohsiung Medical University, No. 100, Tzyou 1st Road, Kaohsiung 807, Taiwan; ^6^Institute of Biomedical Sciences, National Sun Yat-Sen University, Kaohsiung, Taiwan

## Abstract

Artocarpin, a natural prenylated flavonoid, has been shown to have various biological properties. However, its effects on human cutaneous squamous cell carcinoma (SCC) have not been previously investigated. We set out to determine whether artocarpin has cytotoxic effects on SCC cells and whether its pharmacological activity is dependent on protein-nutrient concentration. Our results showed that treatment of HSC-1 cells (a human cutaneous SCC cell line) with artocarpin decreased cell viability and induced cell apoptosis by increasing caspase 3/7 activity. These effects were more pronounced at low fetal bovine serum (FBS) concentrations. Artocarpin induced an increase in the level of phospho-p38 and a decrease in the levels of phospho-ERK, phospho-JNK, phospho-Akt, phospho-mTOR, and phospho-S6K. High FBS concentrations in the culture media inhibited and delayed the uptake of artocarpin from the extracellular compartment (culture media) into the intracellular compartment, as determined by high performance liquid chromatography (HPLC) analysis. In conclusion, artocarpin induces apoptosis in HSC-1 cells through modulation of MAPK and Akt/mTOR pathways. Binding of artocarpin to proteins in the FBS may inhibit cellular uptake and reduce the cytotoxic activity of artocarpin on HSC-1 cells. Therefore, artocarpin may have potential use in the future as a form of treatment for cutaneous SCC.

## 1. Introduction

Cutaneous squamous cell carcinoma (SCC) is a common form of skin cancer which arises from epidermal keratinocytes [1]. The most important risk factor for the development of SCC is ultraviolet radiation from sunlight exposure, especially in people with white skin and those who work outdoors [2]. This skin tumor is locally invasive and may metastasize to regional lymph nodes and visceral organs (including liver, bones, lungs, and brain). Cutaneous SCC is usually treated by surgery [3, 4]. However, surgery is invasive and can cause scarring and disfigurement. Moreover, surgery is not suitable for all patients, particularly elderly patients, patients with underlying medical diseases, those with aggressive tumors which have invaded vital structures, and patients with multiple lesions. In addition, patients with metastatic SCC may require radiotherapy and chemotherapy, which may produce severe side effects [5]. There is therefore a need for development of new and more effective forms of treatment for cutaneous SCC.

**Figure 1:**
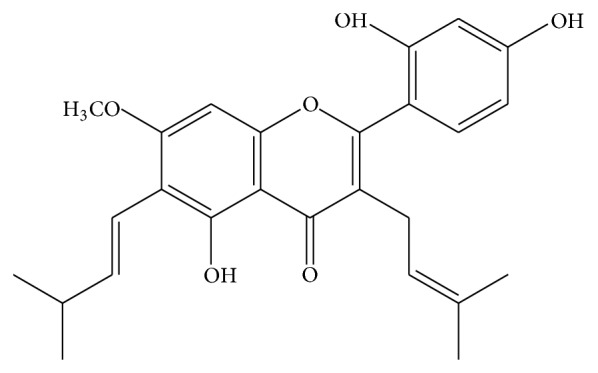
Chemical structure of artocarpin (molecular formula: C_26_H_28_O_6_).

Artocarpin, a prenylated flavonoid (Figure 1), is found in certain agricultural plants, including various species of* Artocarpus* [6]. We have recently demonstrated that artocarpin reduced ultraviolet B-induced skin damage [7] and have antihepatoma activity [8]. We have also shown that extracts of* Artocarpus communis* have antimelanogenesis effects [9]. In addition, previous studies have shown that artocarpin has multiple pharmacological properties, including skin-whitening effects [10–14], inhibition of 5*α*-reductase [15], and cancer cell cytotoxic [16, 17], anti-inflammatory [18], antioxidant [19, 20], and antibacterial activities [21]. To the best of our knowledge, the effects of artocarpin on cutaneous SCC and possible dependence of its cytotoxic activity on protein-nutrient concentration (fetal bovine serum (FBS) concentration) have not been previously investigated.

In this study, we seek to determine whether artocarpin has cytotoxic or apoptotic effects on cutaneous SCC cells and whether this is mediated by the mitogen-activated protein kinase (MAPK) or Akt/mTOR signal transduction pathways. In addition, we evaluate whether the concentration of FBS in the culture media may influence the cellular uptake and cytotoxic activity of artocarpin.

## 2. Materials and Methods

### 2.1. Cells and Reagents

The human cutaneous SCC cell line HSC-1 was obtained from Japanese Collection of Research Bioresources Cell Bank (Osaka, Japan) and was cultured in Dulbecco's Modified Eagle's Medium with 20% FBS. Cells were incubated at 37°C with humidified air containing 5% CO_2_.

Artocarpin was purchased from Pulin Biotech Company Limited (Taipei, Taiwan). The purity of artocarpin was determined to be greater than 98% by high performance liquid chromatography (HPLC) analysis. Phospho-ERK, phospho-p38, and phospho-S6K antibodies were obtained from Cell Signaling Technology (Danvers, MA, USA); phospho-JNK antibody was purchased from Santa Cruz Biotechnology (Dallas, TX); phospho-Akt antibody was from Millipore (Billerica, MA, USA); phospho-mTOR and GAPDH antibodies were from Epitomics (Burlingame, CA, USA).

### 2.2. Alamar Blue Cell Viability Assay

The Alamar Blue assay was used to evaluate cell viability. HSC-1 cells were seeded in 96-well microtiter plates, at a density of 5000 cells per well. After different times of incubation, 10 *μ*L of Alamar Blue (Invitrogen, Carlsbad, CA, USA) was added to each well. Two hours later, the fluorescence of each well was measured using FLx800 fluorescence microplate reader (Bio-Tek, Winooski, VT, USA).

### 2.3. Assessment of Apoptosis by Caspase 3/7 Assay

Apoptosis was assessed using the Apo-ONE Homogeneous Caspase 3/7 Assay Kit (Promega, Madison, WI, USA). HSC-1 cells were seeded in black 96-well plates, at a density of 5000 cells per well. After artocarpin treatment, the caspase substrate Z-DEVD-R110 (rhodamine 110, bis-(N-CBZ-L-aspartyl-L-glutamyl-L-valyl-L-aspartic acid amide)) was diluted to 1 in 100 times in Homogeneous Caspase Buffer to obtain the Homogeneous Caspase 3/7 Reagent. The Homogeneous Caspase 3/7 Reagent was then added to each well and mixed using a plate shaker at room temperature. One hour later, the plates were read on a fluorescence microplate reader.

### 2.4. Western Blotting

HSC-1 cells were cultured in 6-well plates till near confluency. After artocarpin treatment for different periods of time, the cells were harvested and total cellular proteins were extracted with lysis buffer. The BCA Protein Assay Kit (Pierce, USA) was used to estimate the protein content. Proteins were resolved using 10% sodium dodecyl sulfate-polyacrylamide gel electrophoresis (SDS-PAGE) and then transferred onto nitrocellulose membranes. The nitrocellulose membrane was incubated overnight with primary antibodies against phospho-ERK, phospho-p38, phospho-JNK, phospho-Akt, phospho-mTOR, and phospho-S6K, followed by incubation with horseradish peroxidase-conjugated secondary antibody for 1 hour at room temperature. Bound antibodies were detected using an enhanced chemiluminescence system (Millipore). Anti-GAPDH antibody was employed to confirm equal loading of protein between wells.

### 2.5. Measurement of Artocarpin Content in HSC-1 Cells (Intracellular Compartment) and Culture Media (Extracellular Compartment) by HPLC

HSC-1 cells were cultured in different concentrations of FBS (0.2% to 20%) and treated with 10 *μ*M artocarpin for 1, 2, 4, 6, 12, and 24 hours. After artocarpin treatment, the cell lysates (intracellular compartment) and culture media (extracellular compartment) were collected and diluted with methanol. The artocarpin content in the two different compartments was determined by high performance liquid chromatography (HPLC) with ultraviolet (UV) detector system (L-2100, Hitachi, Japan). HPLC was carried out using the LiChroCART 250-4 Purospher STAR RP-18 endcapped (5 *μ*m) column (Merck Millipore, Billerica, MA, USA) at room temperature. The mobile phase was composed of methanol and water (9 : 1), the flow rate was set at 1.0 mL/min, and the wavelength of detection was 282 nm. The artocarpin content in the cell lysates and culture media were identified by comparing the retention time with the artocarpin standard and calculated with the artocarpin standard curve.

### 2.6. Statistical Analysis

The data were presented as mean ± standard error of the mean (SEM). For comparisons of continuous data from two groups, the two-sample *t*-test was used. One-way analysis of variance (ANOVA) was performed to compare continuous data from multiple groups, and Dunnett's test was employed to compare different treatment groups with an untreated control group. Prism software (Graphpad, CA, USA) was used for statistical analysis. Results were considered to be statistically significant if the *P* value was <0.05. All experiments were performed at least three times.

## 3. Results

### 3.1. Artocarpin Induced Cell Death in HSC-1 Cells and Its Cytotoxic Activity Is Dependent on FBS Concentration

**Figure 2:**
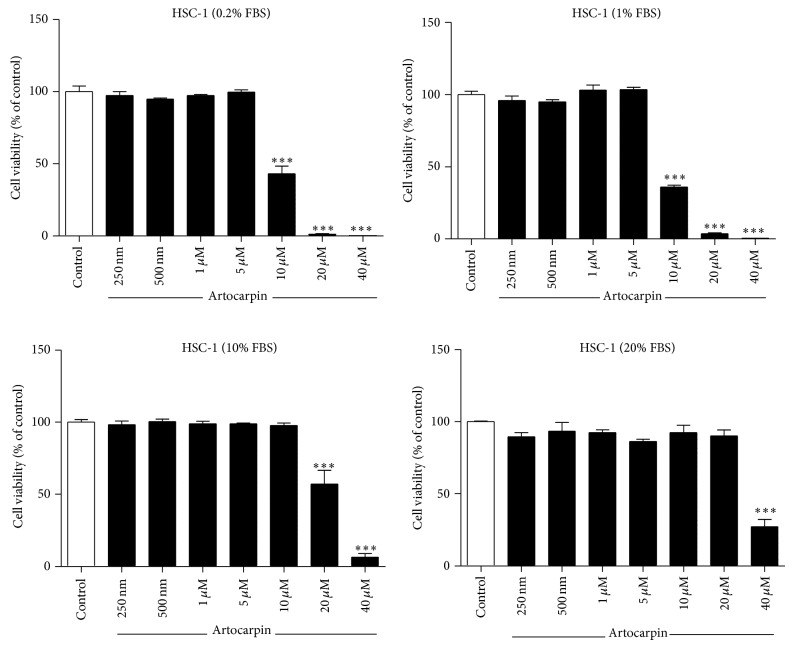
Effect of artocarpin on HSC-1 cell viability when cultured in media containing different concentrations of FBS. Cell viability was determined by the Alamar Blue method 24 hours after addition of artocarpin. Results are shown as mean ± SEM. Data are representative of 3 independent experiments with 4 replicates in each. ^***^ *P* < 0.001 versus untreated control (white bar).

HSC-1 cells were treated with different concentrations of artocarpin for 24 hours and 48 hours, and cell viability was determined by the Alamar Blue assay. When cells were cultured in media containing low concentrations of FBS (0.2% and 1%), artocarpin induced HSC-1 cell death at concentrations of 10 *μ*M and above (Figure 2). When cells were cultured in media containing 10% FBS, artocarpin caused HSC-1 cell death at concentrations of 20 *μ*M and above. At 20% FBS, artocarpin induced HSC-1 cell death only at concentrations above 40 *μ*M. Therefore, the cytotoxic activity of artocarpin was critically dependent on the FBS concentration.

### 3.2. Artocarpin Induced Caspase 3 and 7 Activation in HSC-1 Cells in a Time- and FBS-Dependent Manner

**Figure 3:**
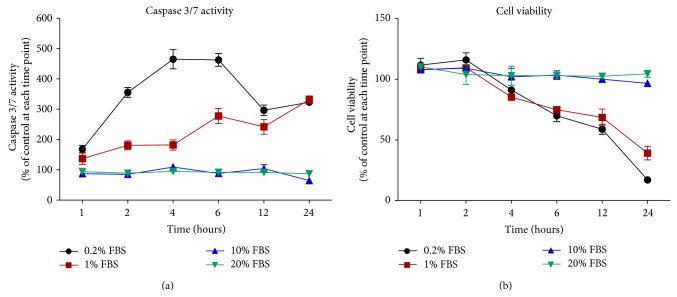
Time-dependent effects of artocarpin (10 *μ*M) on (a) HSC-1 cell apoptosis (determined by the caspase 3/7 assay) and (b) cell viability (determined by the Alamar Blue assay) when cultured in different concentrations of FBS. For each and every time point, caspase 3/7 activity and cell viability were measured for the control group (not treated with artocarpin) and the treatment group (treated with artocarpin), and data were expressed as the ratio (%) between the treatment group and the control group (for the same time point). Results are shown as mean ± SEM. Data are representative of 3 independent experiments with 3 replicates in each.

Cell apoptosis was evaluated with the caspase 3/7 assay. HSC-1 cells were cultured in different concentrations of FBS and treated with 10 *μ*M artocarpin for different periods of time. For each and every time point (1, 2, 4, 6, 12, and 24 hours), caspase 3/7 activity was measured for the control group (not treated with artocarpin) and the treatment group (treated with artocarpin), and data were expressed as the ratio between the treatment group and the control group (for the same time point). When HSC-1 cells were cultured in media containing low concentrations of FBS (0.2% and 1%), treatment with artocarpin induced an increase in caspase 3/7 activity (compared to untreated controls at the same time point) starting at 2 hours and peaking at 4–6 hours (Figure 3(a)). On the other hand, when HSC-1 cells were cultured in media containing high concentrations of FBS (10% and 20%), treatment with 10 *μ*M artocarpin had no significant effect on caspase 3/7 activity (compared to untreated controls at the same time point). Therefore, HSC-1 cells were more sensitive to the apoptotic effects of artocarpin when cultured in low FBS conditions.

Parallel experiments to evaluate cell viability were performed using the Alamar Blue assay. For each and every time point (1, 2, 4, 6, 12, and 24 hours), cell viability was measured for the control group (not treated with artocarpin) and the treatment group (treated with artocarpin), and data were expressed as the ratio between the treatment group and the control group (for the same time point). Our results showed that, at low FBS conditions (0.2% and 1%), artocarpin treatment decreased HSC-1 cell viability (compared to untreated controls at the same time point) starting at 4 hours (Figure 3(b)). Therefore, the increase in caspase 3/7 activity preceded the decrease in cell viability. In contrast, artocarpin had no significant effect on cell viability at high FBS conditions (10% and 20%).

### 3.3. Artocarpin Treatment Induced Changes in the MAPK and Akt/mTOR Pathways in HSC-1 Cells

**Figure 4:**
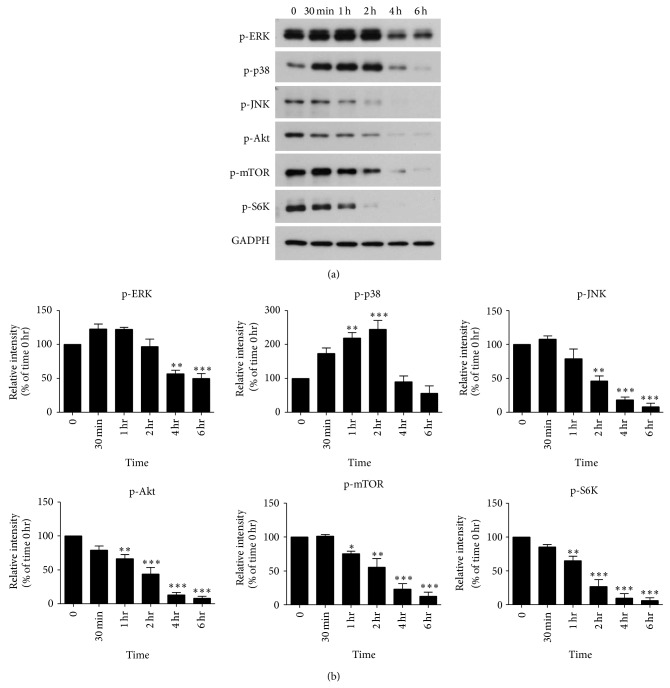
(a) Artocarpin-induced changes in the phosphorylation status of ERK, p38, JNK, Akt, mTOR, and S6K in HSC-1 cells, as determined by Western blotting. Whole cell extracts were prepared after artocarpin (10 *μ*M) treatment for the times shown. The blots are representative of three independent experiments. (b) The relative protein expression levels were quantified by densitometric scanning of immunoblots after adjusting for GAPDH. Data are mean ± SEM of 3 independent experiments. ^*^ *P* < 0.05, ^**^ *P* < 0.01, and ^***^ *P* < 0.001 as compared with the time 0 hr group.

It is well known that cytotoxic agents can induce apoptosis in cancer cells by modulation of MAPK and Akt/mTOR signaling cascades. We, therefore, used Western blotting to determine the effects of artocarpin (10 *μ*M) on these signaling pathways in HSC-1 cells when cultured in 1% FBS. With regard to the MAPK pathway, we found that treatment of HSC-1 cells with artocarpin led to increased levels of phospho-p38 but decreased levels of phospho-ERK and phospho-JNK in a time-dependent manner (Figure 4).

In terms of Akt/mTOR signaling, addition of artocarpin to HSC-1 cells resulted in decreased levels of phospho-Akt, phospho-mTOR, and phospho-S6K in a time-dependent manner (Figure 4). Taken together, our results indicate that treatment with artocarpin led to upregulation of p38 and downregulation of ERK, JNK, and Akt/mTOR pathways in HSC-1 cells.

### 3.4. The Relative Distribution of Artocarpin in the Intracellular and Extracellular Compartments Is Dependent on FBS Concentration

**Figure 5:**
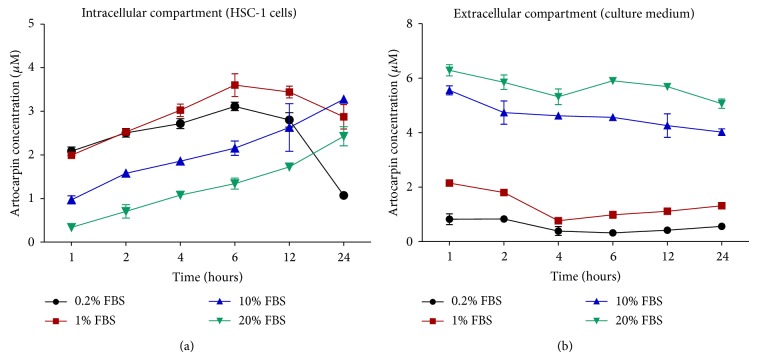
Time-dependent changes in the concentration of artocarpin in (a) HSC-1 cells (intracellular compartment) and (b) culture medium (extracellular compartment) under different concentrations of FBS. Artocarpin concentration in the two different compartments was determined by HPLC-UV analysis after treatment of cells with artocarpin (10 *μ*M) for different periods of time. Results are mean ± SEM of 3 independent experiments.

HSC-1 cells were cultured in media containing different concentrations of FBS and were treated with 10 *μ*M artocarpin for different periods of time. The cell lysates (intracellular compartment) and culture media (extracellular compartment) were harvested, and the concentrations of artocarpin in the two different compartments at different time points were determined by HPLC-UV analysis. The results showed that the relative distribution of artocarpin in the intracellular and extracellular compartments was critically dependent on the FBS concentration. At low FBS conditions (0.2% and 1%), most of the artocarpin was found in the intracellular compartment (Figure 5(a)), especially at earlier time points (1–6 hours), while only small amounts of artocarpin were measured in the extracellular compartment (culture media) (Figure 5(b)). At later time points (after 6 hours), the amount of artocarpin inside cells decreased, possibly as a result of cell death.

On the other hand, at high FBS conditions (10% and 20%), most of the artocarpin was found in the extracellular compartment, while only small amounts of artocarpin were found in the intracellular compartment, especially at earlier time points (1–6 hours). It was only at later time points (after 6 hours) that the amount of artocarpin inside cells gradually rose. These results indicate that high concentrations of FBS in the culture media inhibited and delayed the cellular uptake of artocarpin by HSC-1 cells.

## 4. Discussion

Artocarpin is a prenylated flavonoid derived from* Artocarpus* plants. The chemical structure of artocarpin contains lipophilic isoprenoid groups, which increases its affinity to biological membranes [22]. Recently, artocarpin has been shown to exhibit cytotoxic activity against cancer cells. It was found to be cytotoxic to breast cancer cells by inducing apoptosis [16]. However, the effects of artocarpin on human cutaneous SCC cells have not been previously reported.

The results of the present study showed that artocarpin exhibits cytotoxic effects in HSC-1 cells, and this is mediated by increased caspase 3/7 activity and induction of apoptosis. This suggests that artocarpin may have potential use in the future as a form of treatment for cutaneous SCC, either as topical therapy (applied directly to SCC skin lesions) or as systemic therapy (oral or intravenous) for metastatic SCC. In addition, our results also demonstrated that the sensitivity of SCC cells to the cytotoxic effect of artocarpin is dependent on the FBS concentration. At low FBS conditions (0.2% and 1%), artocarpin shows cytotoxic activity against HSC-1 cells at a concentration of 10 *μ*M, while, at high FBS conditions (such as 20%), artocarpin is only effective at concentrations above 40 *μ*M. Therefore, artocarpin becomes less effective in inducing HSC-1 cell death at higher FBS concentrations. We also demonstrated that artocarpin-induced HSC-1 cell apoptosis is an early event, with the increase in caspase 3/7 activity being apparent as early as 2 hours and cell death occurring after 4 hours of treatment. Previously, a number of flavonoid compounds derived from natural plant sources have also been shown to exhibit cytotoxic activity in certain types of cancers [23, 24].

In terms of signal transduction pathways, we found that treatment of HSC-1 cells with artocarpin induces significant changes in the MAPK and Akt/mTOR pathways. MAPKs are serine/threonine protein kinases involved in the regulation of gene expression, cell proliferation, apoptosis, migration, and differentiation [25, 26]. Humans express three main groups of MAPKs: extracellular signal-regulated kinase- (ERK-) 1/2, p38 proteins, and c-Jun NH_2_-terminal kinases (JNK). Whereas ERK responds primarily to mitogenic stimuli and usually mediates cell growth and survival, p38 is activated by various forms of cellular stress and usually induces cell apoptosis [27]. JNK has been variably found to be proapoptotic or antiapoptotic depending on cell type and experimental systems used [28, 29]. In this study, we found that treatment with artocarpin leads to activation of p38 and suppression of ERK in HSC-1 cells, which is consistent with the drug's apoptotic effects.

Moreover, the Akt/mTOR pathway is a key regulator of cell proliferation, survival, migration, and angiogenesis in human cells. In this signal transduction pathway, the binding of growth factors to their receptors induces the activation of phosphoinositide 3 kinase (PI3K) and Akt, which may then lead to sequential phosphorylation and activation of mTOR and S6K [30–32]. Normally, the activity of this signaling pathway needs to be decreased in order for apoptosis to occur. Our results showed that treatment of HSC-1 cells with artocarpin leads to suppression of Akt, mTOR, and S6K, which is consistent with the induction of apoptosis.

In this study, we also found that the FBS concentration can make a crucial difference to how HSC-1 cells respond to artocarpin and that HSC-1 cells are most sensitive to the cytotoxic and apoptotic effects of artocarpin when the FBS concentration is low. We next set out to determine the reason why the concentration of FBS has such a significant impact on the cytotoxic activity of artocarpin. According to the determination of artocarpin content using HPLC-UV analysis, it was found that the relative distribution of artocarpin in the intracellular and extracellular compartments was dependent on the FBS concentration. At low FBS conditions (0.2% and 1%), most of the artocarpin was found inside HSC-1 cells (intracellular compartment), especially at earlier time points (1–6 hours). At later time points (after 6 hours), the amount of artocarpin inside cells decreased, possibly as a result of cell death. In contrast, at high FBS conditions (10% and 20%), most of the artocarpin was found in the culture media (extracellular compartment). FBS contains mainly bovine serum albumin as well as various growth factors. It is possible that binding of artocarpin with albumin and other proteins in FBS may prevent cellular uptake by and interaction with HSC-1 cells, which may explain the decreased cytotoxic activity of artocarpin at high FBS conditions. Other flavonoids such as quercetin have also been shown to bind with high affinity to serum albumin, which may inhibit cellular uptake of the drug [33, 34].

These findings may have clinical implications, in which the efficacy of artocarpin against cancer cells may be dependent on the extent to which it binds to proteins in blood plasma. A drug in blood may exist in either a bound form (bound to proteins) or an unbound form. Normally, it is the free unbound drug which shows pharmacologic activity [35]. If artocarpin were to be used as systemic treatment for cutaneous SCC, its effects on SCC cancer cells may be affected by plasma protein concentration. In patients taking high protein diet, plasma protein (particularly albumin) levels may be elevated, and the effects of artocarpin may be diminished by binding of drug to plasma proteins. Therefore, dietary protein restriction may be advisable in selected patients in order to enhance the cytotoxic effects of artocarpin. Conversely, the amount of plasma albumin is decreased in the elderly and in those having pathological conditions such as malnutrition, liver disease, renal disease, burns, and surgery [36, 37]. These patients may be more sensitive to the pharmacological effects of artocarpin, and careful monitoring of free drug concentration in the plasma may be required. Recently, human and animal studies have demonstrated that dietary protein restriction is associated with a reduction in cancer growth, progression, and mortality [38–40], which implies that reduced protein intake may be beneficial in a subset of cancer patients. The effects of protein nutrition on cancer development, progression, and response to therapy are intriguing topics that will require further investigation.

Furthermore, it should be noted that plasma protein binding may limit the potential use of artocarpin as a form of systemic treatment. Although long-term dietary protein restriction may decrease plasma albumin and protein levels and increase the amount of free unbound artocarpin available for cellular uptake, an excessively low albumin concentration may be associated with undesirable adverse effects in the clinical setting. Therefore, it is conceivable that artocarpin may have greater potential to be developed as a form of topical treatment rather than systemic therapy for cutaneous SCC.

The results of the present study have shown that artocarpin induces apoptosis in HSC-1 cells through modulation of MAPK and Akt/mTOR pathways. We have also demonstrated that the effect of artocarpin on HSC-1 cells is critically dependent on FBS concentration, and artocarpin entry into HSC-1 cells is decreased and delayed under high FBS conditions. Further studies are warranted to explore the therapeutic potential of artocarpin, particularly as a topical cytotoxic agent for cutaneous SCC.
